# Supplementary material for: An assessment of turnaround times of infant Deoxyribonucleic acid–Polymerase Chain Reaction testing and the associated factors in Western Kenya: A mixed methods study
Source: PLoS One. 2024 May 2;19(5):e0302396. doi: 10.1371/journal.pone.0302396 (PMC11065280; doi:10.1371/journal.pone.0302396)
Supplement: S1 Text — (DOCX) [file pone.0302396.s003.docx]

**Appendix 7: Key Informant Guide**

**Participants: County and sub-county laboratory officers, Laboratory-in charges, MCH/PMTCT coordinators**

1. What is the Ministry of Health protocol or algorithm in EID? Briefly explain.
   1. What protocol is used in your county, sub-county or health facility, if different from MoH?
2. What is the EID laboratory networking in place, if any? Explain
   1. How does it work?
   2. What is the DBS-PCR turnaround time (TAT)?
   3. Is it effective and efficient?
   4. What are the successes if any?
   5. What are the challenges/failure, if any?
3. What are the key programmatic and management recommendations would you suggest?
4. Any other comments
