# Supplementary material for: An assessment of turnaround times of infant Deoxyribonucleic acid–Polymerase Chain Reaction testing and the associated factors in Western Kenya: A mixed methods study
Source: PLoS One. 2024 May 2;19(5):e0302396. doi: 10.1371/journal.pone.0302396 (PMC11065280; doi:10.1371/journal.pone.0302396)
Supplement: S2 Text — (DOCX) [file pone.0302396.s004.docx]

KEY INFORMANT KEYS

| KIIs | KEYS |
| --- | --- |
| Laboratory in-charge in Kambiri health centre in Kakamega County | 1 |
| Laboratory in-charge in Kwhisero health Centre in Kakamega County | 2 |
| Laboratory in-charge at Iguhu health Centre in Kakamega | 3 |
| Bungoma County Laboratory In-Charge | 4 |
| Laboratory in-charge in Busia County referral hospital | 5 |
| Laboratory in-charge at Alupe KEMRI Reference Laboratory | 6 |
| Laboratory-in charges in Emuhaya subcounty | 7 |
| Laboratory in-charge at Hamisi Subcounty hospital in Vihiga | 8 |
| Laboratory-in charges in Sirisia subcounty | 9 |

HEALTH FACILITY KEYS

| HEALTH FACILITY | KEYS |
| --- | --- |
| Kakamega County Referral Hospital | 1 |
| Bungoma County Referral Hospital | 2 |
| Vihiga County Referral Hospital | 4 |
