# Supplementary material for: An assessment of turnaround times of infant Deoxyribonucleic acid–Polymerase Chain Reaction testing and the associated factors in Western Kenya: A mixed methods study
Source: PLoS One. 2024 May 2;19(5):e0302396. doi: 10.1371/journal.pone.0302396 (PMC11065280; doi:10.1371/journal.pone.0302396)
Supplement: Attachment — Submitted filename: Review 17Jan24.docx [file pone.0302396.s006.docx]

Review

Thank you for the opportunity to review this manuscript that reports important data. The data is from 10 yrs ago and so would be better reported if it included the current context of EID in Kenya. Has anything changed since then? Is there any POC EID? Have the stats improved or worsened? Many of the references are very outdated and if used, as they were contemporaneous at the time, should also include more up to date references as discussed specifically below.

POC EID has been accepted and then recommended by the WHO in 2017 and 2021 <https://www.who.int/publications/i/item/WHO-HIV-2017.16> , <https://www.who.int/publications/i/item/9789240022232> so a little more on this methodology and it’s benefits should be discussed rather than one sentence as a recommendation.

This manuscript does not have line numbering as requested by the submission guidelines which makes it difficult to review.

Abstract

The statement “AIDS is beginning to reverse the decades of steady progress….” is no longer true? The child mortality is decreasing in Africa again as PMTCT has been so successful. However infants and children are still dying of HIV which is unacceptable as it treatable and preventable.

Background

Page 3

The first 2 paragraphs has a reference from 2008 and 2007 – this should be updated even if the statistics are similar, as we have to see the relevance of this data for today.

3^rd^ paragraph – remove ‘shortly’ after delivery as it can occur anytime during breastfeeding which can be for years.

Page 4

If you say in 2009 then the last line of the first paragraph should say ‘was beginning to reverse’ – that is historical.

Paragraph 2 first sentence needs updated statistics or report that it was in 2008.

Sentence with reference 7 needs updating as POC EID is now recommended.

Last sentence in the second paragraph is incorrect as there are now many recommended POC technologies.

Paragraph 3 about DBS testing should give dates as is historical.

Page 5

Full paragraph 1 – reference 11 could be updated although statements are true today.

Paragraph 2 – Give the date that this was written 1989 as this is really historical – consider rephrasing - since 1989 it was shown that PRC…..

Paragraph 3 – ‘now expanded’ – when did this happen? And does ‘currently’ refer to 2024?

Page 6 –

Top of page ‘In May 2006’ – has this EID algorithm been updated?

First main paragraph – This is written in the present tense – does this process still happen like this – is there still a CD4 laboratory networking program? Or write it in the past tense.

2^nd^ paragraph – Reference 12 is from 1989! – the 2010 WHO recommended 4 weeks for the EID results to get back to the caregiver <https://www.who.int/publications/i/item/9789241599801>

Page 7

‘many patients never receive their results’ – this also means babies may be undiagnosed with increased mobidity and mortality – and the test is a wasted cost. Receiving the result is not the end point.

‘One challenge is that the type of laboratory…….’ This is only if POC PCR technologies are not used. This should be differentiated.

2^nd^ paragraph – as this was done long ago I suggest putting in the past tense – ‘associated factors had….’ And ‘This study sought to….’

‘The results would also guide….’ – did this happen or is the situation the same currently?

Materials and methods

Sample size – where is a) and there is a b)

Page 8

It would be good to upload the data collection tools and the questionnaires for review

Page 9

Figure 1

Age in infants is commonly given in weeks and would be clearer to interpret with the main spike at 6 weeks as expected.

Page 10

Figure 2 Should Global not be in the title of the figure for clarity?

In the figure or title there needs to be clarity that the times are the mean times. Figures should be stand alone and not need text to interpret.

Discussion

Page 14

I disagree with the first sentence on this page as a negative HIV test is very important for a mother to receive to know her baby is not infected. Please reference or consider that both positive and negative results are important for medical and psychological reasons.

It is believed the timelier the ~~rapid~~ testing is performed. Rapid does not make sense here and can be omitted. Rapid usually refers to a POC test.

Page 16

Paragraph 1

‘The reason for delayed specimen processing could be…..’ were the lab managers who were interviewed asked about this?

Paragraph 3

Bubbing should be hubbing?

As mentioned at the beginning – due to POC EID being recommended by WHO now there should be at least a sentence explaining what it is and how it is better rather than the first mention of it being as a single line recommendation. It solves nearly all the problems of this study.

This is important data to have published and was well collected and analysed.
