# Supplementary material for: An assessment of turnaround times of infant Deoxyribonucleic acid–Polymerase Chain Reaction testing and the associated factors in Western Kenya: A mixed methods study
Source: PLoS One. 2024 May 2;19(5):e0302396. doi: 10.1371/journal.pone.0302396 (PMC11065280; doi:10.1371/journal.pone.0302396)
Supplement: Attachment — Submitted filename: Response to Reviewers.docx [file pone.0302396.s007.docx]

**REBUTTAL LETTER**

| Comments | **Response** |
| --- | --- |
| Journal Requirements: |  |
| 1. When submitting your revision, we need you to address these additional requirements.  Please ensure that your manuscript meets PLOS ONE's style requirements, including those for file naming. The PLOS ONE style templates can be found at  https://journals.plos.org/plosone/s/file?id=wjVg/PLOSOne_formatting_sample_main_body.pdf and  https://journals.plos.org/plosone/s/file?id=ba62/PLOSOne_formatting_sample_title_authors_affiliations.pdf | Noted and revised accordingly. |
| 2. We noticed you have some minor occurrence of overlapping text with the following previous publication(s), which needs to be addressed:  Omondi, M.P., Ombaka, J., Mwau, M. and Ouma, C., 2016. Mother-to-child HIV transmission using single, dual and triple ARV prophylaxis regimens and their correlates in western Kenya: chart review. African Journal of Pharmacology and Therapeutics, 5(1).  In your revision ensure you cite all your sources (including your own works), and quote or rephrase any duplicated text outside the methods section. Further consideration is dependent on these concerns being addressed. | Noted and revised accordingly. |
| 3. We are unable to open your Supporting Information file PCR database.sav. Please kindly revise as necessary and re-upload. | I have re-uploaded and I have been able to open the SPSS dataset with SPSS version 27.0 myself. |
| 4. Please include captions for your Supporting Information files at the end of your manuscript, and update any in-text citations to match accordingly. Please see our Supporting Information guidelines for more information: http://journals.plos.org/plosone/s/supporting-information. | Done |
| **Review** |  |
| Thank you for the opportunity to review this manuscript that reports important data. The data is from 10 yrs ago and so would be better reported if it included the current context of EID in Kenya. Has anything changed since then? Is there any POC EID? Have the stats improved or worsened? Many of the references are very outdated and if used, as they were contemporaneous at the time, should also include more up to date references as discussed specifically below.  POC EID has been accepted and then recommended by the WHO in 2017 and 2021 <https://www.who.int/publications/i/item/WHO-HIV-2017.16> , <https://www.who.int/publications/i/item/9789240022232> so a little more on this methodology and it’s benefits should be discussed rather than one sentence as a recommendation.  This manuscript does not have line numbering as requested by the submission guidelines which makes it difficult to review. | Noted. Substantial additions on POC EID updated with relevant references.  Line numbering inserted. |
| Abstract  The statement “AIDS is beginning to reverse the decades of steady progress….” is no longer true? The child mortality is decreasing in Africa again as PMTCT has been so successful. However infants and children are still dying of HIV which is unacceptable as it treatable and preventable. | Noted. Edited. |
| Background |  |
| Page 3  The first 2 paragraphs has a reference from 2008 and 2007 – this should be updated even if the statistics are similar, as we have to see the relevance of this data for today.  3^rd^ paragraph – remove ‘shortly’ after delivery as it can occur anytime during breastfeeding which can be for years. | Revised  Edited. |
| Page 4  If you say in 2009 then the last line of the first paragraph should say ‘was beginning to reverse’ – that is historical. | Revised |
| Paragraph 2 first sentence needs updated statistics or report that it was in 2008.  Sentence with reference 7 needs updating as POC EID is now recommended.  Last sentence in the second paragraph is incorrect as there are now many recommended POC technologies. | Revised  Revised. Is recommended but currently still not functional due to lack of reagents and the old 2010 laboratory networking of DBS specimens to Alupe KEMRI is very much in place. I would advise the reviewers to contact USAID projects in Kakamega, Vihiga, Bungoma and Busia to confirm the non-functional POCs in the health facilities from the time they were handed over to county government by EGPAF in 2015. Specimen batching and hubbing remains in place to date.  Revised. |
| Paragraph 3 about DBS testing should give dates as is historical. | Noted |
| Page 5  Full paragraph 1 – reference 11 could be updated although statements are true today.  Paragraph 2 – Give the date that this was written 1989 as this is really historical – consider rephrasing - since 1989 it was shown that PRC…..  Paragraph 3 – ‘now expanded’ – when did this happen? And does ‘currently’ refer to 2024? | Revised with updated citations.  Edited.  Edited. It refers to current status in 2014. |
| Page 6 –  Top of page ‘In May 2006’ – has this EID algorithm been updated?  First main paragraph – This is written in the present tense – does this process still happen like this – is there still a CD4 laboratory networking program? Or write it in the past tense.  2^nd^ paragraph – Reference 12 is from 1989! – the 2010 WHO recommended 4 weeks for the EID results to get back to the caregiver <https://www.who.int/publications/i/item/9789241599801> | Edited. We now have the revised 2016 algorithm  This is still the current status. The point –of-care testing has never actually taken off in western Kenya after the piloting phase. It was handed over to the county government who were asked to supply reagents. The county governments have been unable to supply reagents and the old CD4 Lab networking is still in force to date.  Revised. Added the new citation. |
| Page 7  ‘many patients never receive their results’ – this also means babies may be undiagnosed with increased mobidity and mortality – and the test is a wasted cost. Receiving the result is not the end point.  ‘One challenge is that the type of laboratory…….’ This is only if POC PCR technologies are not used. This should be differentiated.  2^nd^ paragraph – as this was done long ago I suggest putting in the past tense – ‘associated factors had….’ And ‘This study sought to….’  ‘The results would also guide….’ – did this happen or is the situation the same currently? | Noted.  Edited.  Edited.  Edited. This unfortunately was not disseminated. I am in contact with USAID project in Kakamega, Vihiga, Bungoma and Busia town for dissemination of these findings once peer reviewed and published. They will organize a dissemination forum – virtual or physical once the article is published. |
| Materials and methods  Sample size – where is a) and there is a b)  Page 8  It would be good to upload the data collection tools and the questionnaires for review  Page 9  Figure 1  Age in infants is commonly given in weeks and would be clearer to interpret with the main spike at 6 weeks as expected.  Page 10  Figure 2 Should Global not be in the title of the figure for clarity?  In the figure or title there needs to be clarity that the times are the mean times. Figures should be stand alone and not need text to interpret. | Edited  Done  Noted. Age was collected in months in this study.  The figure shows other turnaround times other than global TAT. I have added a legend to make the figures stand alone. |
| Discussion |  |
| Page 14  I disagree with the first sentence on this page as a negative HIV test is very important for a mother to receive to know her baby is not infected. Please reference or consider that both positive and negative results are important for medical and psychological reasons.  It is believed the timelier the ~~rapid~~ testing is performed. Rapid does not make sense here and can be omitted. Rapid usually refers to a POC test.  Page 16  Paragraph 1  ‘The reason for delayed specimen processing could be…..’ were the lab managers who were interviewed asked about this?  Paragraph 3  Bubbing should be hubbing?  As mentioned at the beginning – due to POC EID being recommended by WHO now there should be at least a sentence explaining what it is and how it is better rather than the first mention of it being as a single line recommendation. It solves nearly all the problems of this study.  This is important data to have published and was well collected and analysed. | Edited and referenced accordingly.  Edited  No. I didn’t ask them this question to KEMRI Alupe Laboratory staff. I gave an expert opinion on possible reasons since I was working in Western Kenya at the time of the study.  Noted and edited.  Noted. I have included a paragraph on POC EID.  Noted. |
| Reviewer #2: |  |
| The Assessment of Dry Blood Spot –Polymerase Chain Reaction (DBS-PCR) Turnaround Time (TAT) and The Associated Factors in Western Kenya: A Cross-sectional Study  Title: It indicates that this is a cross-sectional study and yet from the description it is a mixed study design. I suggest leaving out the design from the tile or revising it to what it truly is  DBS PCR is not an assay name – the correct assay name is DNA PCR, DBS is a sample type. Consider revising the title and the entire write-up to reflect this  What is the definition of TAT in this study’s context? | Edited accordingly  Revised the title and the entire write-up to reflect this.  Time from specimen receipt to dispatch in the laboratory network cascade. Included in the manuscript. |
| Abstract  AIDS is not reversing progress made in MTCT – so much progress has been made and hence the statement is misleading. Make us of the term HIV exposed infant to refer to the study subjects.  For uniformity, decide on whether you use infant or baby. Choose one to be used throughout the paper.  Recommendation is outdated, so much progress has been made in testing HEIs and therefore this conclusion will not help. Batching is no longer being done, unless due to other reasons but not due to lack of testing capacity. POCs for DNA PCR are also available. | Edited.  Done  Noted. However, batching and hubbing is still being done in the study areas because the POC EID have encountered challenges mostly due to shortages of laboratory reagents. County governments have not absorbed the cost of running the POC EID in the study areas and so Alupe KEMRI remains the testing sites with the attendant laboratory networking as explained in the manuscript. |
| Background  Outdated references have been used in the entire write-up, which does not entirely reflect the current situation for HEI lab testing. There are updated and modern policies and guidelines that need to be referenced here. This entire section needs to be revised with updated information  We have more than 10 testing labs in the country, not 4 as indicated  what specific issues contribute to the long TATs at these study sites? the author only mentions batching and hubbing but is not clear on the specifics | Noted. Significant revisions made. Updated citations. Clarifications made as shown in manuscript with track changes.  Updated. There are 12 testing labs and the 13^th^ one is in Migori and yet to pick up  Noted. |
| Inclusion criteria How did you obtain written IC from a facility? This is not possible since IC only applies to individual/group study subjects. A facility is not a subject  Why did you need mother baby pairs yet the study was retrospective? Did the authors interact directly with study subjects?  Why did you include facilities testing HEI after 2012? | Edited. Administrative approval was obtained.  Mother-baby pairs were useful since this was part of a bigger study that determined the HIV transmission rates. There was no interaction with mothers.  That is the time when we started having accurate and consistent use of MoH HEI registers in these four counties. |
| Consenting County directors cannot provide IC on behalf of facilities, perhaps you mean a letter of approval was provided by the county directors. | Edited. Administrative approval provided by the county health directors and the health facility in-charges. |
| Results What does global TAT mean?  How was the KII done? Where and what tools were used? Where are the demographic and distribution results of the 9KIIs? Please include the methodology, analysis and results for the KII.  Did you look at whether delayed TAT had any impact on the outcome/result? What proportion were positives? | Global TAT is the time from specimen dispatch from health facility and the results received back at the health facility.  Revised.  No. the study did not look at the impact of TAT on outcome. The result on positivity is already published: Omondi, M.P., Ombaka, J., Mwau, M. and Ouma, C., 2016. Mother-to-child HIV transmission using single, dual and triple ARV prophylaxis regimens and their correlates in western Kenya: chart review. African Journal of Pharmacology and Therapeutics, 5(1). |
| Discussion Please update this entire section based on current information on HEI. | Done. |
| Limitation This study was conducted in 2012 – over 10 years ago. This should be a limitation since linking it to the current trends on HEI is challenging | Noted |
