# Supplementary material for: An assessment of turnaround times of infant Deoxyribonucleic acid–Polymerase Chain Reaction testing and the associated factors in Western Kenya: A mixed methods study
Source: PLoS One. 2024 May 2;19(5):e0302396. doi: 10.1371/journal.pone.0302396 (PMC11065280; doi:10.1371/journal.pone.0302396)
Supplement: Attachment — Submitted filename: Response to Reviewers.docx [file pone.0302396.s008.docx]

**REBUTTAL LETTER**

| Comments | **Response** |
| --- | --- |
| Journal Requirements: |  |
| Please review your reference list to ensure that it is complete and correct. If you have cited papers that have been retracted, please include the rationale for doing so in the manuscript text, or remove these references and replace them with relevant current references. Any changes to the reference list should be mentioned in the rebuttal letter that accompanies your revised manuscript. If you need to cite a retracted article, indicate the article’s retracted status in the References list and also include a citation and full reference for the retraction notice. | Noted |
|  |  |
| **Review** |  |
| Reviewer #1: Review of Revision 1 |  |
| Thanks you for the thorough revision of this paper. It is much improved.  Please confirm if it was DNA-PCR as some analysers use RNA-PCR and there is also TNR-PCT (total nucleic acid using both?) If different analysers were used just use PCR testing.  I suggest keeping the sample type so saying PCR testing of dry blood spots where applicable. | Thanks  The analyser in KEMRI Alupe Laboratory uses DNA only.  Noted. |
| Line 26 Title The title still needs Polymerase Chain Reaction written out -DNA? is insufficient. This is through the text as well. Also add Infant in the title so as to specify the testing group Suggest – An assessment of turnaround times of infant Deoxyribonucleic acid – Polymerase Chain Reaction testing and the associated factors in Western Kenya: a mixed methods study | Noted.  Revised accordingly. |
| Line 38 Can abbreviate DNA-PCR throughout if DNA is accurate or just use PCR and comment. | Noted. |
| Line 41 DNA-PCR turnaround time (TAT) of dry blood spots (DBS) – important to mention the sample type and write out TAT in full as it is the first time mentioned. Then TAT abbreviation thereafter, as in line 47 | Noted. |
| Line 43 Can delete design – mixed methods study (methods should be plural throughout) | Noted. |
| Line 55 The TAT for DNA-PCR specimens | Noted. |
| Line 61 Suggest EID is another key word | Added |
| Line 67 and Line 90 have different 2022 stats of the number of children with HIV? The sources are different UNAIDS 0-14 and Unicef 0-19 so choose which one and specify ages. Suggest UNAIDS as 10-19 are adolescents and not relevant to this publication? | Revised. |
| Results Line 342 Mean (singular) number of days | Revised. |
| Discussion  Line 441 TAT definition is not needed as mentioned more clearly above.  It is disappointing to hear the POC testing has not been supported as this is the future of diagnostic laboratory medicine. We continue to build up evidence and implementation practice to turn this tide. All the best in Kenya. | Noted  Noted |
